# Prediction of Acute Kidney Injury after Liver Transplantation: Machine Learning Approaches vs. Logistic Regression Model

**DOI:** 10.3390/jcm7110428

**Published:** 2018-11-08

**Authors:** Hyung-Chul Lee, Soo Bin Yoon, Seong-Mi Yang, Won Ho Kim, Ho-Geol Ryu, Chul-Woo Jung, Kyung-Suk Suh, Kook Hyun Lee

**Affiliations:** 1 Department of Anesthesiology and Pain Medicine, Seoul National University Hospital, Seoul 03080, Korea; azong@hanmail.net (H.-C.L.); yunsb0107@gmail.com (S.B.Y.); seongmi.yang@gmail.com (S.-M.Y.); hogeol@gmail.com (H.-G.R.); jungcwoo@gmail.com (C.-W.J.); leekh@snu.ac.kr (K.H.L.); 2 Department of Anesthesiology and Pain Medicine, Seoul National University College of Medicine, Seoul 03080, Korea; 3 Department of Surgery, Seoul National University Hospital, Seoul National University College of Medicine, Seoul 03080, Korea; kssuh@snu.ac.kr

**Keywords:** acute kidney injury, liver transplantation, machine learning

## Abstract

Acute kidney injury (AKI) after liver transplantation has been reported to be associated with increased mortality. Recently, machine learning approaches were reported to have better predictive ability than the classic statistical analysis. We compared the performance of machine learning approaches with that of logistic regression analysis to predict AKI after liver transplantation. We reviewed 1211 patients and preoperative and intraoperative anesthesia and surgery-related variables were obtained. The primary outcome was postoperative AKI defined by acute kidney injury network criteria. The following machine learning techniques were used: decision tree, random forest, gradient boosting machine, support vector machine, naïve Bayes, multilayer perceptron, and deep belief networks. These techniques were compared with logistic regression analysis regarding the area under the receiver-operating characteristic curve (AUROC). AKI developed in 365 patients (30.1%). The performance in terms of AUROC was best in gradient boosting machine among all analyses to predict AKI of all stages (0.90, 95% confidence interval [CI] 0.86–0.93) or stage 2 or 3 AKI. The AUROC of logistic regression analysis was 0.61 (95% CI 0.56–0.66). Decision tree and random forest techniques showed moderate performance (AUROC 0.86 and 0.85, respectively). The AUROC of support the vector machine, naïve Bayes, neural network, and deep belief network was smaller than that of the other models. In our comparison of seven machine learning approaches with logistic regression analysis, the gradient boosting machine showed the best performance with the highest AUROC. An internet-based risk estimator was developed based on our model of gradient boosting. However, prospective studies are required to validate our results.

## 1. Introduction

Analytics for predicting postoperative morbidity has been limited to the classical statistic techniques, such as logistic regression analysis and the Cox proportional hazard model. However, these models require the statistical assumption of the independent and linear relationship between explanatory and outcome variables. Furthermore, limitations of overfitting and multicollinearity of the regression analysis preclude the analysis of a large number of variables. These limitations have made prediction models to select a small number of variables that are known to be clinically relevant.

Recently, novel machine learning techniques have demonstrated improved predictive performance compared to classical statistical methods limited to logistic regression. For example, there have been reports of using machine learning techniques to predict postoperative clinical outcomes including specific morbidity or in-hospital mortality [1,2,3]. Compared to the logistic regression or Cox proportional hazard model, reports using machine learning techniques have shown lower prediction error. For acute kidney injury, previous studies demonstrated that machine learning techniques have excellent performance or better performance compared to logistic regression analysis in hospitalized patients [4] or patients undergoing major surgery [5]. However, although previous studies used different techniques of machine learning, including neural network [1,2], random forest [3], support vector machine [5], and gradient boosting machine [4], a performance comparison among these specific techniques of machine learning has rarely been conducted.

Postoperative acute kidney injury (AKI) is an important complication after liver transplantation which is associated with poor graft survival and increased mortality [6,7,8,9]. To find risk factors and develop a risk prediction model, many studies have reported using classical regression methods [6,10,11,12,13,14,15]. Although several risk factors have been identified [6,7,8,9,10,11,12,13,14,15], their performance was rarely reported [6] regarding the area under the receiver-operating characteristics curve (AUROC) [13], which is the primary measure of the prediction model [16]. Furthermore, previous studies did not include a sufficient number of variables due to multi-collinearity. In addition, the possible non-linear relationship between explanatory variables and the outcome variable could not be fully considered. However, machine learning techniques are relatively free of these limitations of conventional statistical analysis and may demonstrate better performance. If machine learning techniques have a better performance to predict AKI, risk prediction at the end of surgery could be possible with readily-available patient data from electronic medical records.

Therefore, firstly, we attempted to compare the performance of the prediction of acute kidney injury after liver transplantation by machine learning techniques with the prediction by multivariable logistic regression. We hypothesized that the prediction by machine learning techniques may have better performance than logistic regression. Secondly, we sought to compare the performance of difference machine learning techniques used in the previous studies at the same time. Techniques including gradient boosting machine, random forest, decision tree, support vector machine, naïve Bayes, neural network, and deep belief network were used in our analysis. Third, we planned to build a risk estimator which could be used in the daily practice based on our best prediction model by machine learning technique.

## 2. Materials and Methods

### 2.1. Study Design

This retrospective observational study was approved by the institutional review board of Seoul National University Hospital (1805-137-948). We retrospectively reviewed the electronic medical records of 1398 consecutive patients, who underwent living donor liver transplantation (LDLT) or deceased donor liver transplantation (DDLT) at our institution between November 2004 and December 2015. Informed consent was waived because of the study’s retrospective design. Pediatric cases (*n* = 152) and those with missing baseline serum creatinine (*n* = 35) were excluded and the remaining 1211 cases were analyzed.

### 2.2. Anesthesia and Surgical Techniques

Anesthesia was induced with propofol and maintained with sevoflurane, remifentanil, and rocuronium. Volume controlled ventilation was maintained with a tidal volume of 6–8 mL/kg. Arterial-line catheters were inserted into the radial and femoral arteries. A pulmonary artery catheter was inserted routinely, and continuous cardiac index and right ventricle-associated variables were monitored. Ephedrine and continuous infusion of dopamine and/or norepinephrine and/or epinephrine were used to treat hypotension according to the monitored cardiac index, mixed venous oxygen saturation (SvO_2_) and systemic vascular resistance (SVR).

Donor grafts were prepared with a histidine-tryptophan-ketoglutarate solution. The piggyback technique was used to anastomose the graft and donor vessels. End-to-end anastomosis of the hepatic artery and duct-to-duct anastomosis of the bile duct were performed in succession. During surgery, immunosuppression was induced with basiliximab 20 mg (Simulect, Novartis Pharma B.V., Arnhem, The Netherlands) and methylprednisolone 500 mg (Solumedrol, Pfizer, Ballerup, Denmark). Postoperative immunosuppression was initiated with a calcineurin inhibitor of tacrolimus with mycophenolate mofetil on the first postoperative day.

### 2.3. Data Collection

Based on the previous literature, data related to demographic or perioperative variables known to be related to postoperative renal dysfunction were collected from the institutional electronic medical record (Table 1) [6,7,10,13,17]._ENREF_17_ENREF_14_ENREF_18_ENREF_19. Preoperatively, the Model for End-stage Liver Disease (MELD) score, the Child–Turcotte–Pugh (CTP) score, and the Child–Pugh classification were determined for all patients [18]. Data on patient demographics, history of hypertension, diabetes mellitus, other baseline medical history, ABO blood type incompatibility, baseline laboratory findings including preoperative serum albumin were collected. Data on surgery and anesthesia-related variables including warm and cold ischemic time, graft-recipient body-weight ratio (GRWR), intraoperative estimated blood loss, intraoperative transfusion, amount of infused crystalloid, hydroxyethyl starch, and albumin levels were collected. Data on intraoperative laboratory and hemodynamic variables were collected. Hemodynamic variables included mean arterial pressure, mean pulmonary arterial pressure, central venous pressure, SvO_2_, cardiac index, and SVR. These hemodynamic variables were collected at the following eight time points: after anesthesia induction, 1 h after the end of anesthesia induction when pulmonary artery catheter was inserted, 10 min after the beginning of the anhepatic phase, 5 min before and after graft reperfusion, 20 min after reperfusion, 5 min after the completion of biliary reconstruction, and at the end of surgery. These hemodynamic variables were then averaged and entered into the analysis.

The primary outcome variable was postoperative AKI defined by acute kidney injury network (AKIN) criteria. Postoperative AKI was determined based on the maximal change in sCr level during first two postoperative days [19]. The most recent sCr measured before surgery was used as a baseline. The AKIN serum creatinine criteria are shown in Supplemental Table S1. Urine output criteria were not used because different cutoff of oliguria may be required for AKI after surgery [20,21]. Stage 2 or 3 AKI were used as secondary outcomes because higher stages of AKI is more strongly associated with patient mortality and stage 1 AKI may be only functional and transient [22].

### 2.4. Statistical Analysis

SPSS software version 23.0 (IBM Corp., Armonk, NY, USA) and Python programming language (Python Software Foundation, version 3.5.2) were used for our analysis. The following packages for machine learning were used: Scikit-learn (https://github.com/scikit-learn/scikit-learn), XGboost (https://github.com/dmlc/xgboost) [23,24], and Keras (https://github.com/keras-team/keras). Scikit-learn package was used for logistic regression, decision tree, random forest, naïve Bayes, and support vector machine. XGboost was used for gradient boosting machine. Keras was used for neural network and deep belief network.

A total of 72 explanatory variables including the variables in Table 1 were used for machine learning. Before developing prediction models, our collected data were divided into 70% of training dataset cases and 30% of test dataset cases. The cases in the training dataset were used for developing machine learning and logistic regression model. The cases in the testing dataset was used for validating and comparing the performance of the models developed in the training dataset. Each machine learning method had its own hyperparameters such as the number of layers in neural network or number of trees in random forest. To find the optimal hyperparameters, a 10-fold cross-validation was used. This cross-validation process was used only for developing the model and performance of the developed final models was evaluated in the testing dataset. All possible combinations of hyperparameters were investigated by grid search (a list of investigated hyperparameters are provided in Supplemental Text S1. The hyperparameters with the highest average validation AUROC were considered as optimal hyperparameters. After that, the final model of each technique was re-fitted with optimal hyperparameters and the entire training dataset. The test dataset was used only for testing the final model’s performance.

In the neural network model and the support vector machine, the values of each variable were normalized using the mean and standard deviation of the training dataset. The maximum and minimum values of the training dataset were used for scaling values between 0 and 1 in the deep belief network. In the logistic regression, naïve Bayes and tree models, normalization was not performed because it was not necessary.

A multivariable logistic regression analysis including variables in Table 1 was performed to identify independent predictors that established a multivariable prediction model. The variables that were closely correlated were excluded before the multivariable analysis—e.g., MELD score and preoperative serum albumin level—to avoid multicollinearity. Backward stepwise variable selection was used using a cutoff of *p* < 0.10. As a sensitivity analysis, logistic regression analysis was performed without stepwise variable selection.

Missing data were present in <5% of records. We imputed the missing values according to the incidence of missing. If the incidence of missing was <1%, the missing was substituted by the mean for continuous variable and by the mode for incidence variable. Missing values of variables with a ratio of missing was >1% and <5% were replaced by hot-deck imputation, where a missing value was imputed from a randomly selected value of the variable. Our primary analysis attempted to compare the prediction ability of machine learning approaches to logistic regression model in terms of AUROC [25]. We also compared the accuracy, which was defined as the sum of the number of cases with true positive and true negative results divided by total number of test set.

Finally, a risk estimator based on our model with the best performance (https://vitaldb.net/aki_liver). This estimator calculates the risk of developing AKI after liver transplantation (from 0 to 1 value) and classifies the risk into three classes of the low, moderate, and high risk of AKI.

## 3. Results

A total of 1211 cases including 367 (30.3%) deceased donor and 844 (69.7%) living donor liver transplantation were included in our analysis. During the first two postoperative days, AKI, as determined by AKIN criteria, was observed in 365 patients (30.1%), and stage 2 or 3 AKI developed in 76 patients (6.3%). The incidence of AKI was 26.1% (220/844) for LDLT patients and 39.5% (145/367) for DDLT patients. The incidence of stage 2 or 3 AKI was 5.3% (45/844) for LDLT and 8.4% (31/367) for DDLT. Patient demographics and surgery and anesthesia-related variables in both training and test set are presented in Table 1.

The optimal hyperparameters found in a 10-fold cross-validation and the accuracy and AUROCs of the test set were shown in Table 2. Gradient boosting machine showed the largest test AUROC (0.90, 95% confidence interval [CI] 0.86–0.93) and highest accuracy (84%). The deep belief network classifier showed the smallest test AUROC (0.59) with the low accuracy (65%). The results of logistic regression analysis with and without stepwise variable selection are shown in Table 3 and Table S2. Seven variables were selected as independent predictors. AUROC of the multivariable logistic prediction model for the test dataset was 0.61 (95% confidence interval [CI] 0.56 to 0.66).

**Figure 1:**
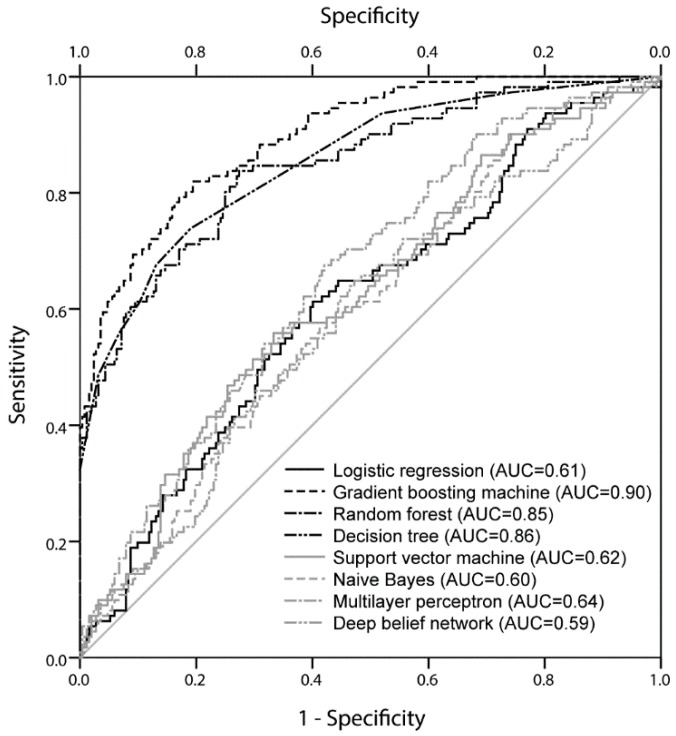
Comparison of area under the receiver operating characteristic curves among the machine learning models and the logistic regression model to predict acute kidney injury of all stages. AUC = area under the receiver operating characteristic curve.

Table 3 and Figure 1 show the comparison of test AUROC to predict AKI of all stages according to the model. The AUROC of gradient boosting machine was significantly larger than for all other models (AUROC 0.90, 95% CI 0.86 to 0.93, all *p* < 0.001). The AUROC of the decision tree and random forest techniques showed better performance than that of other models but worse than the gradient boosting machine. The AUROCs of the support vector machine, naïve Bayes, neural network, and deep belief network were similar to that of logistic regression model but significantly smaller than that of other machine learning models (*p* < 0.001). Supplemental Table S3 shows the comparison test of the AUROC of our models to predict stage 2 or 3 AKI. The AUROC of the gradient boosting machine was also significantly larger than the AUROCs of all the other models, except random forest.

**Figure 2:**
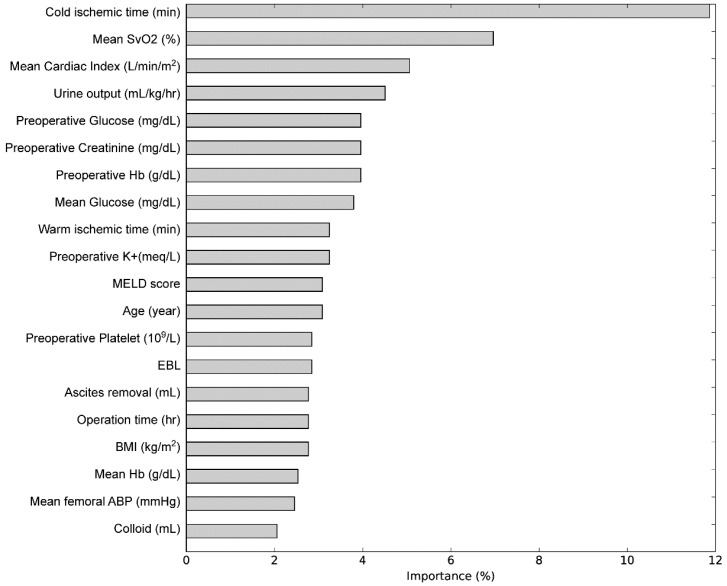
Variance importance plot of the gradient boosting machine. SvO_2_ = mixed venous oxygen saturation; Hb = hemoglobin; MEDL = model for end-stage liver disease; EBL = estimated blood loss; BMI = body-mass index; ABP = arterial blood pressure.

**Figure 3:**
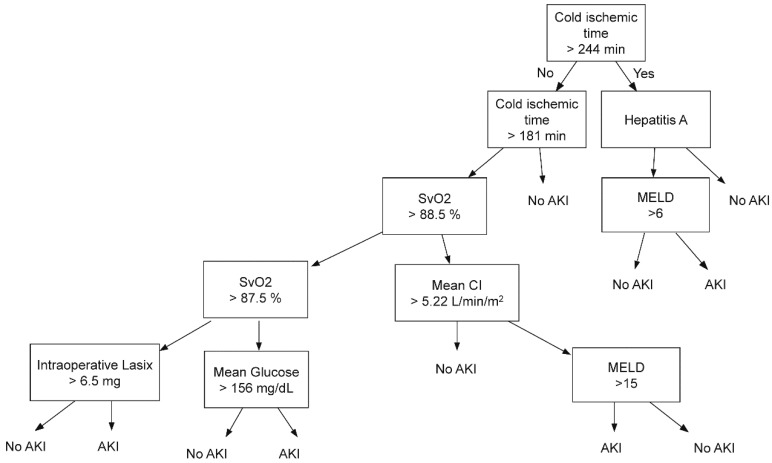
Decision tree showing the classification of patients with and without acute kidney injury. AKI = acute kidney injury; SvO_2_ = mixed venous oxygen saturation; MEDL = model for end-stage liver disease.

The importance matrix plot for gradient boosting machine is shown in Figure 2 and cold ischemic time and intraoperative mean SvO_2_ were ranked first and second. The stepwise binary classification criteria of the best decision tree model with a depth of 5 are shown in Figure 3.

## 4. Discussion

We compared the predictive ability of 7 machine learning models and a logistic regression model to predict AKI after liver transplantation. The result showed that gradient boosting machine has the largest AUROC and highest accuracy to predict both AKI of all stages and stage 2 or 3 AKI. The authors previously reported the superior predictive ability by the gradient boosting for AKI after cardiac surgery [26]. Our study results are consistent with the previous study with different analysis tool. We also developed an internet-based risk estimator based on our gradient boosting model. This estimator should be prospectively validated for its clinical use to determine the risk of AKI at the end of liver transplantation surgery. Further prospective multicenter trials are required to validate the better performance of gradient boosting.

For the prediction of a non-linear relationship, the gradient boosting machine builds a sequential series of decision trees, where each tree corrects the residuals in the predictions made by the previous tress. After each step of boosting, the algorithm scales the newly added weights, which balances the influence of each tree. In addition, the gradient boosting machine uses techniques that aim to reduce overfitting by only a random subset of descriptors in building a tree [23]. The impact of gradient boosting machine has been recognized in a number of machine learning and data mining challenges. Our results also demonstrated that the gradient boosting machine appears to be a very effective and efficient machine learning method.

Although its predictive ability was less than that of the gradient boosting machine, the performance of random forest was also better than that of logistic regression in our dataset. Random forest is an extension of traditional decision tree classifiers with an ensemble technique [27]. Each tree is constructed using a random subset of the original training data and a random subset of the explanatory variables. Random forest can minimize overfitting by making the decision by voting of these randomly generated trees [28]. However, this advantage of random forests seems much more effective when many variables and a large number of datasets are used for learning. The results of the present study showed no significant performance gain over the simple decision tree model.

Simple decision tree analysis showed a good performance in our study. A decision tree is a hierarchical model that recursively splits the dataset based on the Gini impurity or entropy [29,30,31]. A decision tree could have better prediction ability than logistic regression model under certain circumstances because it uses different variable and threshold in every branch. The greatest advantage of the decision tree model is that it gives interpretable decision rules after training. However, the deeper the depth of the tree, the more difficult it is to interpret, and the risk of overfitting also increases.

Although multilayer perceptron showed a good performance to predict in-hospital mortality in a previous study [1], the performance of the neural network and deep belief network in our study was inferior to those of all other machine learning techniques. The reason for this may be explained by the fact that the relationship between the explaining variables and outcome variable is largely non-linear. Although multilayer perceptron is able to approximate any nonlinear function, a large number of learning data are required. Therefore, our dataset might be not large enough to train the multilayer perceptron [32].

There have been some reports that performance of the machine learning techniques are not superior to conventional risk score or the logistic regression model to predict mortality [1,33]. However, in these studies, they compared the performance to predict in-hospital mortality in a study sample with a low incidence (<1%) [1,33]. Therefore, the difference might not be statistically proven. In this study, it could be compared easily with a higher incidence of the outcome variable (30.3%).

Cold ischemic time and intraoperative mean SvO_2_ were considered to be the most important variables to classify the development of AKI by the gradient boosting machine and decision tree [34,35]. This may be attributed to the fact that our study sample consisted of mixed populations including both DDLT and LDLT patients. The wide variability in cold ischemic time between deceased donor and living donor and a higher incidence of AKI in DDLT may have produced the high discriminative power of cold ischemic time. Therefore, cold ischemic time may be less important for prediction of AKI after either DDLT or LDLT. A low SvO_2_ suggests poor oxygen delivery to the major organs including the kidney [36]. During liver transplantation, clamping of inferior vena cava and intraoperative bleeding decrease cardiac output and major organ perfusion, resulting in poor oxygen delivery, especially during graft reperfusion [37]. Therefore, SvO_2_ may be an important hemodynamic goal. Further prospective trials may evaluate the effect of the optimizing intraoperative SvO_2_ to reduce the risk of AKI after liver transplantation.

Our study has several limitations. First, our analysis used only a small number of cases from data derived from an Asian single-center with mixed living and deceased donor transplantation. The performance of machine learning techniques might be different when they are applied to a sample of different institution with a different distribution of covariates. The race difference might be a significant predictor of AKI, which could not be evaluated in our dataset. External validity of our prediction model may be limited. By machine learning approach using each institution’s specific data set for training, each institution could obtain their own specific model best fit for their institution. We uploaded the source code for learning the gradient boosting model used in this study as a Supplemental Text S2. Therefore, each institution may develop their own prediction model by machine learning approach using their historical electronic medical record data and update their model periodically. The real-time processing of patient data would yield risk prediction for each patient after surgery. Second, the results from our machine learning models are more difficult to interpret than the results from the logistic regression model [3]. However, gradient boosting machine and decision tree provided for the modest interpretability through the variance importance plot and the inspection of the decision rule in tree nodes. Third, it is not certain that our results could translate into improved clinical outcomes for the patients undergoing liver transplantation. Many of our variables of importance are not clinically modifiable, and accurate risk prediction may not be followed by improved patient outcomes. However, a further prospective trial should evaluate whether the adjustment of hemodynamic variables including SvO_2_ could decrease the incidence of AKI.

## 5. Conclusions

In conclusion, our study demonstrated that a machine learning model with gradient boosting machine, random forest, and decision tree showed better performance than the traditional logistic regression model to predict AKI after liver transplantation. Among these models, the gradient boosting machine showed the best performance with the highest AUROC. An internet-based risk estimator was developed based on our gradient boosting model. This estimator could help a clinician to predict AKI at the end of surgery. Since gradient boosting machines can be used in real-time prediction, further studies are required to prospectively validate our results and improve clinical outcomes after liver transplantation.

## Figures and Tables

**Table 1:** Patient characteristics in this study.

	Training Dataset (*n* = 848)	Testing Dataset (*n* = 363)	*p*-Value
AKI defined by AKIN criteria (*n*)	254 (30%)	111 (31%)	0.882
AKI AKIN stage (*n*)			
No AKI	594 (70%)	252 (69%)	0.846
Stage 1 AKI	198 (23%)	91 (25%)	
Stage 2 AKI	42 (5%)	15 (4%)	
Stage 3 AKI	14 (2%)	5 (1%)	
Demographic data			
Age, recipient (years)	54.0 (48.0–60.0)	53.0 (48.0–60.0)	0.747
Gender (female)	224 (26%)	116 (32%)	0.058
Body mass index (kg/m^2^)	23.1 (20.9–25.3)	23.1 (21.4–25.3)	0.466
Surgery type			
Deceased donor (*n*)	265 (31%)	105 (29%)	0.461
ABO incompatibility (*n*)	26 (3%)	14 (4%)	0.596
Medical history			
Hypertension (*n*)	92 (11%)	38 (10%)	0.924
Diabetes mellitus (*n*)	125 (15%)	53 (15%)	0.980
Ischemic heart disease (*n*)	17 (2%)	4 (1%)	0.388
Chronic kidney disease (*n*)	63 (7%)	26 (7%)	0.966
Cerebrovascular accident (*n*)	8 (1%)	5 (1%)	0.714
COPD (*n*)	19 (2%)	5 (1%)	0.446
Pulmonary hypertension (*n*)	10 (1%)	7 (2%)	0.454
Prolonged QT interval (*n*)	33 (4%)	9 (2%)	0.290
Preoperative medication			
Insulin (*n*)	43 (5%)	10 (3%)	0.099
Beta blocker (*n*)	37 (4%)	18 (5%)	0.760
Diuretics (*n*)	26 (3%)	22 (6%)	0.022
Cause of liver transplantation			
Hepatitis B (*n*)	355 (42%)	137 (38%)	0.203
Hepatocellular carcinoma (*n*)	383 (45%)	178 (49%)	0.240
Alcoholic liver cirrhosis (*n*)	85 (10%)	40 (11%)	0.675
Hepatitis C (*n*)	61 (7%)	30 (8%)	0.597
Hepatitis A (*n*)	4 (0%)	2 (1%)	1.000
Acute hepatic failure (*n*)	54 (6%)	22 (6%)	0.942
Cholestatic liver cirrhosis (*n*)	21 (2%)	7 (2%)	0.709
Metabolic cause (*n*)	4 (0%)	4 (1%)	0.250
Preoperative status			
MELD score	15 (12–22)	15 (12–22)	0.635
Child–Turcotte–Pugh score	8 (6–10)	8 (6–10)	0.979
Child–Turcotte–Pugh class			
Class 1	253 (30%)	98 (27%)	0.571
Class 2	331 (39%)	144 (40%)	
Class 3	264 (31%)	121 (33%)	
Hepato-renal syndrome (*n*)	138 (16%)	44 (12%)	0.078
Pleural effusion (*n*)	55 (6%)	30 (8%)	0.324
Spontaneous bacterial peritonitis (*n*)	46 (5%)	30 (8%)	0.082
Esophageal variceal ligation (*n*)	181 (21%)	90 (25%)	0.213
Hepatic encephalopathy (*n*)	109 (13%)	46 (13%)	0.994
Trans-arterial chemoembolization (*n*)	200 (24%)	79 (22%)	0.538
Portal hypertension (*n*)	44 (5%)	26 (7%)	0.225
Previous operation history (*n*)	368 (43%)	158 (44%)	0.983
Preoperative measurements			
LVEF (%)	65 (62–68)	65 (62–69)	0.645
Hemoglobin (g/dL)	10.9 (9.2–12.6)	10.7 (9.35–12.3)	0.599
Albumin (g/dL)	3.0 (2.5–3.5)	3.0 (2.6–3.4)	0.593
Creatinine (mg/dL)	0.90 (0.74–1.17)	0.90 (0.73–1.10)	0.485
Platelet (10^9^/L)	64 (47–95)	64 (45–89)	0.233
Na^+^ (mEq/L)	137 (132–140)	137 (132–140)	0.771
K^+^ (mEq/L)	4.1 (3.8–4.4)	4.1 (3.8–4.5)	0.324
Glucose (mg/dL)	103 (89–133)	103 (90–131)	0.766
Surgery and anesthesia details			
Operation time (h)	6.83 (5.78–7.92)	6.75 (5.65–8.0)	0.348
Anesthesia time (h)	7.92 (6.92–9.0)	7.92 (6.67–9.0)	0.376
Cold ischemic time (min)	86 (67–240)	86 (66–230)	0.460
Warm ischemic time (min)	30 (28–35)	30 (26–35)	0.227
GRWR < 0.8 (*n*)	45 (5%)	16 (4%)	0.609
Ascites removal (mL)	0 (0–2000)	0 (0–2000)	0.490
Use of Intraoperative CRRT	26 (3%)	9 (2%)	0.711
Use of Intraoperative venovenous bypass	20 (2%)	4 (1%)	0.225
Estimated blood loss (mL)	3000 (1550–6150)	2930 (1500–6000)	0.867
Urine output (mL/kg/h)	0.93 (0.58–1.55)	0.91 (0.51–1.60)	0.694
Intraoperative fluid management			
Crystalloid (L)	3.5 (2.45–5.2)	3.6 (2.6–5.3)	0.404
Colloid (mL)	0 (0–500)	0 (0–500)	0.915
Albumin (mL)	300 (100–400)	300 (100–400)	0.948
Intraoperative transfusion			
Red blood cell transfusion (unit)	6.0 (2.0–12.0)	6.0 (2.0–12.0)	0.854
Fresh frozen plasma transfusion (unit)	6.0 (1.0–12.0)	6.0 (0.0–12.0)	0.634
Platelet transfusion (unit)	0.0 (0.0–6.0)	0.0 (0.0–6.0)	0.705
Cryoprecipitate transfusion (unit)	0.0 (0.0–0.0)	0.0 (0.0–0.0)	0.287
Intraoperative drugs			
Dose of epinephrine, bolus (ug)	6.5 (0.0–20.0)	10.0 (0.0–25.0)	0.127
Dose of furosemide, bolus (mg)	0.0 (0.0–5.0)	0.0 (0.0–5.0)	0.965
Use of dopamine, continuous (*n*)	160 (19%)	55 (15%)	0.142
Use of epinephrine, continuous (*n*)	23 (3%)	2 (1%)	0.028 *
Use of norepinephrine, continuous (*n*)	38 (4%)	14 (4%)	0.737
Intraoperative measurements			
Mean SvO_2_ (%)	89 (87–90)	89 (87–90)	0.981
Mean CVP (mmHg)	6 (5–8)	7 (5–8)	0.913
Mean femoral ABP (mmHg)	69 (62–75)	69 (62–75)	0.790
Mean cardiac index (L/min/m^2^)	4.24 (3.86–4.86)	4.24 (3.84–4.77)	0.418
Mean hemoglobin (g/dL)	9.3 (8.4–10.6)	9.3 (8.2–10.3)	0.110
Mean blood glucose (mg/dL)	162 (145–179)	162 (143–181)	0.973

* *p*-value < 0.05. Data are presented as median (interquartile range) or number (%). AKI = acute kidney injury; AKIN = acute kidney injury network; COPD = chronic obstructive pulmonary disease; MELD = Model for End-stage Liver Disease; LVEF = left ventricular ejection fraction; GRWR = graft-recipient body-weight ratio; CRRT = continuous renal replacement therapy; SvO_2_ = mixed venous oxygen saturation; CVP = central venous pressure; ABP = arterial blood pressure.

**Table 2:** Comparison of area under receiver-operating characteristic curve among the different models to predict acute kidney injury of all stages.

	Optimal Hyperparameter	AUROC (95% CI)	Accuracy	*p*-Value
Logistic regression (LR)		0.61 (0.56–0.66)	0.68	<0.001 vs. GBM <0.001 vs. RF <0.001 vs. DT 0.670 vs. SVM 0.608 vs. NB 0.239 vs. MLP 0.414 vs. DBN
Gradient boosting machine (GBM)	Maximum depth = 5 Number of estimators = 100, gamma = 0.4	0.90 (0.86–0.93)	0.84	0.001 vs. RF 0.033 vs. DT <0.011 vs. SVM <0.001 vs. NB <0.001 vs. MLP <0.001 vs. DBN
Random forest (RF)	Maximum depth = 5 Number of estimators = 150	0.85 (0.81–0.89)	0.80	0.918 vs. DT <0.001 vs. SVM <0.001 vs. NB <0.001 vs. MLP <0.001 vs. DBN
Decision tree (DT)	Maximum depth = 5 Criterion = Gini index	0.86 (0.81–0.89)	0.81	<0.001 vs. SVM <0.001 vs. NB <0.001 vs. MLP <0.001 vs. DBN
Support vector machine (SVM)	Kernel = radial basis C = 1.0 Log(gamma) = −3	0.62 (0.57–0.67)	0.69	0.287 vs. NB 0.300 vs. MLP 0.084 vs. DBN
Naive Bayes (NB)	Model = Gaussian	0.60 (0.54–0.65)	0.64	0.088 vs. MLP 0.701 vs. DBN
Multilayer perceptron (MLP)	Number of hidden layers = 2 Number of nodes in a layer = 8	0.64 (0.59–0.69)	0.66	0.016 vs. DBN
Deep belief network (DBN)	Number of hidden layers = 2 Number of nodes in a layer = 8	0.59 (0.53–0.64)	0.65	

CI = confidence interval; AUROC = area under the receiver operating characteristic curve.

**Table 3:** Results of multivariable logistic regression analysis for acute kidney injury.

Variable	Beta-Coefficient	Odds Ratio	95% CI	*p*-Value
Child–Turcotte–Pugh score	0.067	1.069	0.999–1.144	0.055
GRWR less than 0.8	0.669	1.952	1.021–3.733	0.043
Operation time (per hour)	0.384	1.472	1.008–2.149	0.045
Cold ischemic time (per 30 min)	0.147	1.159	1.092–1.230	<0.001
Transfusion of red blood cells (per 1 unit)	0.017	1.017	1.002–1.031	0.022
Intraoperative colloid administration (per 500 mL)	0.269	1.309	1.119–1.531	0.001
Intraoperative urine output (mL/kg/h)	−0.156	0.856	0.730–1.003	0.054
Intraoperative mean SvO_2_ decrease (per 10%)	0.311	1.285	1.099–1.501	0.002
Intraoperative mean blood glucose level (per 1 mg/dL)	0.081	1.085	1.029–1.144	0.003

Multivariable logistic regression analysis was performed using all the variables with *p* < 0.2 in univariate logistic analysis. Stepwise backward variable selection process was used for this analysis using a cutoff of *p*-value of less than 0.10. Nagelkerke R^2^ statistic was 0.163. Hosmer and Lemeshow goodness of fit test was not significant at 5% (*p* = 0.701). GRWR = graft-recipient body-weight ratio, SvO_2_ = mixed venous oxygen saturation.

## Supplementary Materials

The following are available online at http://www.mdpi.com/2077-0383/7/11/428/s1, Supplemental Text S1: Investigated hyperparameter in each model; Supplemental Text S2: Python source code for learning the gradient boosting model used in our study; Supplemental Table S1: AKIN (acute kidney injury network) serum creatinine diagnostic criteria of acute kidney injury; Supplemental Table S2: Results of multivariable logistic regression analysis for acute kidney injury without stepwise variable selection; Supplemental Table S3: Comparison of area under receiver-operating characteristic curve among the different models for predicting stage 2 or 3 acute kidney injury.

## References

[1] Lee C.K., Hofer I., Gabel E., Baldi P., Cannesson M. (2018). Development and Validation of a Deep Neural Network Model for Prediction of Postoperative in-hospital Mortality. Anesthesiology.

[2] Fei Y., Hu J., Li W.Q., Wang W., Zong G.Q. (2017). Artificial neural networks predict the incidence of portosplenomesenteric venous thrombosis in patients with acute pancreatitis. J. Thromb. Haemost..

[3] Taylor R.A., Pare J.R., Venkatesh A.K., Mowafi H., Melnick E.R., Fleischman W., Hall M.K. (2016). Prediction of In-hospital Mortality in Emergency Department Patients with Sepsis: A Local Big Data-Driven, Machine Learning Approach. Acad. Emerg. Med..

[4] Koyner J.L., Carey K.A., Edelson D.P., Churpek M.M. (2018). The Development of a Machine Learning Inpatient Acute Kidney Injury Prediction Model. Crit. Care Med..

[5] Thottakkara P., Ozrazgat-Baslanti T., Hupf B.B., Rashidi P., Pardalos P., Momcilovic P., Bihorac A. (2016). Application of Machine Learning Techniques to High-Dimensional Clinical Data to Forecast Postoperative Complications. PLoS ONE.

[6] Utsumi M., Umeda Y., Sadamori H., Nagasaka T., Takaki A., Matsuda H., Shinoura S., Yoshida R., Nobuoka D., Satohm D. (2013). Risk factors for acute renal injury in living donor liver transplantation: Evaluation of the RIFLE criteria. Transpl. Int..

[7] Lebron Gallardo M., Herrera Gutierrez M.E., Seller Perez G., Curiel Balsera E., Fernandez Ortega J.F., Quesada Garcia G. (2004). Risk factors for renal dysfunction in the postoperative course of liver transplant. Liver Transpl..

[8] Barri Y.M., Sanchez E.Q., Jennings L.W., Melton L.B., Hays S., Levy M.F., Klintmalm G.B. (2009). Acute kidney injury following liver transplantation: Definition and outcome. Liver Transpl..

[9] Thomas M.E., Blaine C., Dawnay A., Devonald M.A., Ftouh S., Laing C., Latchem S., Lewington A., Milford D.V., Ostermann M. (2015). The definition of acute kidney injury and its use in practice. Kidney Int..

[10] Chen J., Singhapricha T., Hu K.Q., Hong J.C., Steadman R.H., Busuttil R.W., Xia V.M. (2011). Postliver transplant acute renal injury and failure by the RIFLE criteria in patients with normal pretransplant serum creatinine concentrations: A matched study. Transplantation.

[11] Paugam-Burtz C., Kavafyan J., Merckx P., Dahmani S., Sommacale D., Ramsay M., Belghiti J., Mantz J. (2009). Postreperfusion syndrome during liver transplantation for cirrhosis: Outcome and predictors. Liver Transpl..

[12] Vives M., Callejas R., Duque P., Echarri G., Wijeysundera D.N., Hernandez A., Sabate A., Bes-Rastrollo M., Monedero P. (2016). Modern hydroxyethyl starch and acute kidney injury after cardiac surgery: A prospective multicentre cohort. Br. J. Anaesth..

[13] Park M.H., Shim H.S., Kim W.H., Kim H.J., Kim D.J., Lee S.H., Kim C.S., Gwak M.S., Kim G.S. (2015). Clinical Risk Scoring Models for Prediction of Acute Kidney Injury after Living Donor Liver Transplantation: A Retrospective Observational Study. PLoS ONE.

[14] Jun I.G., Kwon H.M., Jung K.W., Moon Y.J., Shin W.J., Song J.G., Hwang G.S. (2018). The Impact of Postreperfusion Syndrome on Acute Kidney Injury in Living Donor Liver Transplantation: A Propensity Score Analysis. Anesth. Analg..

[15] Jun I.G., Lee B., Kim S.O., Shin W.J., Bang J.Y., Song J.G., Song G.W., Lee S.G., Hwang G.S. (2016). Comparison of acute kidney injury between ABO-compatible and ABO-incompatible living donor liver transplantation: A propensity matching analysis. Liver Transpl..

[16] Zou K.H., O’Malley A.J., Mauri L. (2007). Receiver-operating characteristic analysis for evaluating diagnostic tests and predictive models. Circulation.

[17] Hilmi I.A., Damian D., Al-Khafaji A., Sakai T., Donaldson J., Winger D.G., Kellum J.A. (2015). Acute kidney injury after orthotopic liver transplantation using living donor versus deceased donor grafts: A propensity score-matched analysis. Liver Transpl..

[18] Selzner M., Kashfi A., Cattral M.S., Selzner N., McGilvray I.D., Greig P.D., Levy G.A., Renner E.L., Grant D.R. (2010). Live donor liver transplantation in high MELD score recipients. Ann. Surg..

[19] Cockcroft D.W., Gault M.H. (1976). Prediction of creatinine clearance from serum creatinine. Nephron.

[20] Hori D., Katz N.M., Fine D.M., Ono M., Barodka V.M., Lester L.C., Yenokyan G., Hogue C.W. (2016). Defining oliguria during cardiopulmonary bypass and its relationship with cardiac surgery-associated acute kidney injury. Br. J. Anaesth..

[21] Mizota T., Yamamoto Y., Hamada M., Matsukawa S., Shimizu S., Kai S. (2017). Intraoperative oliguria predicts acute kidney injury after major abdominal surgery. Br. J. Anaesth..

[22] Kellum J.A., Zarbock A., Nadim M.K. (2017). What endpoints should be used for clinical studies in acute kidney injury?. Intens. Care Med..

[23] Sheridan R.P., Wang W.M., Liaw A., Ma J., Gifford E.M. (2016). Extreme Gradient Boosting as a Method for Quantitative Structure-Activity Relationships. J. Chem. Inf. Model..

[24] Gao C., Sun H., Wang T., Tang M., Bohnen N.I., Muller M., Herman T., Giladi N., Kalinin A., Spino C. (2018). Model-based and Model-free Machine Learning Techniques for Diagnostic Prediction and Classification of Clinical Outcomes in Parkinson’s Disease. Sci. Rep..

[25] DeLong E.R., DeLong D.M., Clarke-Pearson D.L. (1988). Comparing the areas under two or more correlated receiver operating characteristic curves: A nonparametric approach. Biometrics.

[26] Lee H.-C., Yoon H.-K., Nam K., Cho Y., Kim T., Kim W., Bahk J.H. (2018). Derivation and Validation of Machine Learning Approaches to Predict Acute Kidney Injury after Cardiac Surgery. J. Clin. Med..

[27] Li K., Yu N., Li P., Song S., Wu Y., Li Y., Liu M. (2017). Multi-label spacecraft electrical signal classification method based on DBN and random forest. PLoS ONE.

[28] Hsu P.L., Robbins H. (1947). Complete Convergence and the Law of Large Numbers. Proc. Natl. Acad. Sci. USA.

[29] Aviles-Jurado F.X., Leon X. (2013). Prognostic factors in head and neck squamous cell carcinoma, comparison of CHAID decision trees technology and Cox analysis. Head Neck.

[30] Kasbekar P.U., Goel P., Jadhav S.P. (2017). A Decision Tree Analysis of Diabetic Foot Amputation Risk in Indian Patients. Front. Endocrinol..

[31] Zintzaras E., Bai M., Douligeris C., Kowald A., Kanavaros P. (2007). A tree-based decision rule for identifying profile groups of cases without predefined classes: Application in diffuse large B-cell lymphomas. Comput. Biol. Med..

[32] Hornik K. (1991). Approximation capabilities of multilayer feedforward networks. Neural Netw..

[33] Kuo P.J., Wu S.C., Chien P.C., Rau C.S., Chen Y.C., Hsieh H.Y., Hsieh C.H. (2018). Derivation and validation of different machine-learning models in mortality prediction of trauma in motorcycle riders: A cross-sectional retrospective study in southern Taiwan. BMJ Open..

[34] Kalisvaart M., Schlegel A., Umbro I., de Haan J.E., Scalera I., Polak W.G., IJzermans J.N.M., Mirza D.F., Perera M.T.P.R., Isaac J.I. (2018). The Impact of Combined Warm Ischemia Time on Development of Acute Kidney Injury in Donation After Circulatory Death Liver Transplantation: Stay Within the Golden Hour. Transplantation.

[35] Rudnick M.R., Marchi L.D., Plotkin J.S. (2015). Hemodynamic monitoring during liver transplantation: A state of the art review. World J. Hepatol..

[36] Mayer K., Trzeciak S., Puri N.K. (2016). Assessment of the adequacy of oxygen delivery. Curr. Opin. Crit. Care.

[37] Adelmann D., Kronish K., Ramsay M.A. (2017). Anesthesia for Liver Transplantation. Anesthesiol. Clin..

