# Supplementary material for: Prediction of Acute Kidney Injury after Liver Transplantation: Machine Learning Approaches vs. Logistic Regression Model
Source: J Clin Med. 2018 Nov 8;7(11):428. doi: 10.3390/jcm7110428 (PMC6262324; doi:10.3390/jcm7110428)
Supplement: Supplementary file 1 [file jcm-07-00428-s001.pdf]

Prediction of acute kidney injury after liver transplantation: machine learning approaches *vs.* logistic regression model

Hyung-Chul Lee, Soo Bin Yoon, Seong-Mi Yang, Won Ho Kim, Ho-Geol Ryu, Chul-Woo Jung, Kyung-Suk Suh, Kook Hyun Lee

Supplemental Digital Contents

Supplement Text S1. Investigated hyperparameters in each model

Supplemental Text S2. Python source code for learning the gradient boosting model used in our study.

Supplemental Table S1. AKIN (acute kidney injury network) serum creatinine diagnostic criteria of acute kidney injury used in our study.

Supplemental Table S2. Results of multivariable logistic regression analysis for acute kidney injury of all stages without stepwise variable selection.

Supplemental Table S3. Comparison of area under receiver-operating characteristic curve among the different models to predict stage 2 or 3 acute kidney injury.

Supplement Text S1. Investigated hyperparameters in each model

1) Gradient boosting machine, Random forest
    A. Number of estimators: 10, 30, 50, 80, 100, 150, 200
    B. Maximum depth: 2, 3, 4, 5, 6, 7
2) Decision Tree
    A. Criterion: Gini index, entropy
    B. Maximum depth: 2, 3, 4, 5, 6, 7
3) Support vector machine (linear)
    A. C: 0.1 step from 0.1 to 2.0
4) Support vector machine (radial basis)
    A. C: 0.5 step from 0.5 to 10
    B. Log(gamma): 0.1 step from -5 to 0
5) Multilayer perceptron, Deep belief network
    A. Number of hidden layers: 2, 3, 4, 5, 6
    B. Number of nodes in a hidden layer: 8, 10, 12, 16, 32, 64

Supplemental Text S2. Python source code for learning the gradient boosting model used in our study.

```
import pandas as pd
import xgboost as xgb
import numpy as np
from sklearn.metrics import confusion_matrix, accuracy_score, roc_curve, roc_auc_score, f1_score
import random
from scipy.stats import beta

def roc_ci(y_true, y_pred):
    total = len(y_true)
    success = roc_auc_score(y_true, y_pred) * total
    alpha = 0.05
    lower = beta.ppf(alpha / 2, success, total - success + 1)
    upper = beta.ppf(1 - alpha / 2, success + 1, total - success)
    return lower, upper

nfold = 10
param = {'n_estimators': 100, 'depth':5, 'gamma':0.4}

df = pd.read_csv("data.csv")
y = df.values[:, 0]    # output variable (0/1) in the first column
X = df.values[:, 1:]    # input variables from the second column

nsamp = len(y)
ntest = int(nsamp * 0.3)    # 30% of samples are testing dataset
X_test = X[nsamp-ntest:,:]
y_test = y[nsamp-ntest:]
X_trainval = X[:nsamp-ntest,:]
```

```
y_trainval = y[:nsamp-ntest]

print('{} training, {} testing'.format(nsamp - ntest, ntest))

ntrainval = len(y_trainval)
idx = np.arange(0, ntrainval)
random.shuffle(idx)

aucs = []
models = []
for ifold in range(nfold):
    train_idx = list(idx[:int(ifold * ntrainval / nfold)]) + list(idx[int((1+ifold) * ntrainval / nfold):])
    val_idx = idx[int(ifold * ntrainval / nfold):int((1+ifold) * ntrainval / nfold)]

    X_train = X_trainval[train_idx,:]
    y_train = y_trainval[train_idx]
    y_val = y_trainval[val_idx]
    X_val = X_trainval[val_idx,:]

    if len(np.unique(y_val)) <= 1:
        continue

    model = xgb.sklearn.XGBClassifier(max_depth=param['depth'], n_estimators=param['n_estimators'], gamma=param['gamma'])
    model.fit(X_train, y_train)

    y_pred = model.predict_proba(X_val)[:, 1]
    auc = roc_auc_score(y_val, y_pred)
    fpr, tpr, thvals = roc_curve(y_val, y_pred)
```

```
        y_pred = y_pred > 0.5
        f1 = f1_score(y_val, y_pred)
        acc = accuracy_score(y_val, y_pred)
        tn, fp, fn, tp = confusion_matrix(y_val, y_pred).ravel()
        print('fold {}\tauc {:.3f}\tacc {:.3f}\tF1 {:.3f}\tTN {}\tfp {}\tfn {}\tTP {}'.format(ifold, auc, acc, f1, tn, fp, fn, tp))

        models.append(model)
        aucs.append(auc)

# refit with trainval dat
idx_best = np.argmax(aucs)
print('\nretraining the best model #{} with {} samples'.format(idx_best, len(y_trainval)))
model = models[idx_best]
model.fit(X_trainval, y_trainval)

y_pred = model.predict_proba(X_test)[:, 1].ravel()

# test the final model
auc = roc_auc_score(y_test, y_pred)
lower_bound, upper_bound = roc_ci(y_test, y_pred)
y_pred = y_pred > 0.5
f1 = f1_score(y_test, y_pred)
acc = accuracy_score(y_test, y_pred)
tn, fp, fn, tp = confusion_matrix(y_test, y_pred).ravel()

print('test auc: {:.3f} ({:.3f} - {:.3f})\tacc: {:.3f}\tf1: {:.3f}\tTN {}\tfp {}\tfn {}\tTP {}'.format(auc, lower_bound, upper_bound, acc, f1, tn, fp, fn, tp))
```

Supplemental Table S1. AKIN (acute kidney injury network) serum creatinine diagnostic criteria of acute kidney injury used in our study.

KDIGO criteria	Serum creatinine criteria
Stage 1	Increase in sCr by 0.3 mg/dl or increase in sCr to 1.5-1.9 times baseline within postoperative 48 hours
Stage 2	Increase in sCr to 2.0-2.9 times baseline within postoperative 48 hours
Stage 3	Increase in sCr to > 4.0 mg/dl with an acute increase of >0.5 mg/dL or increase in sCr to 3.0 times baseline or initiation of renal replacement therapy within postoperative 48 hours

sCr = serum creatinine.

Supplemental Table S2. Results of multivariable logistic regression analysis for acute kidney injury of all stages without stepwise variable selection.

Variable	Beta-coefficient	Odds Ratio	95% CI	P-value
Deceased donor	-0.68	0.51	0.13 - 1.94	.320
Recipient age (year)	0.01	1.01	0.99 - 1.03	.297
Recipient gender	0.06	1.06	0.73 - 1.55	.752
Body-mass index (kg/m^2)	0.01	1.01	0.96 - 1.06	.805
Hypertension	-0.33	0.72	0.40 - 1.30	.273
Diabetes mellitus	0.03	1.03	0.59 - 1.80	.918
Angina pectoris	-0.13	0.88	0.28 - 2.77	.828
COPD	-0.08	0.93	0.31 - 2.75	.892
Chronic kidney disease	-0.40	0.67	0.34 - 1.34	.262
Cerebrovascular accident	0.56	1.76	0.37 - 8.25	.476
Pulmonary hypertension	-0.70	0.50	0.09 - 2.90	.437
MELD score	-0.01	0.99	0.97 - 1.02	.442
Child Turcotte Pugh score	0.11	1.11	1.02 - 1.22	.018
ABO incompatibility	-0.52	0.59	0.20 - 1.75	.345
Left ventricular ejection fraction (%)	0.01	1.01	0.98 - 1.04	.618
Preoperative hemoglobin (g/dL)	-0.08	0.93	0.84 - 1.03	.150
Pleural effusion	-0.31	0.73	0.37 - 1.47	.379
Alcoholic cirrhosis	0.01	1.01	0.58 - 1.77	.967
Metabolic cause	0.95	2.58	0.32 - 21.1	.377
Cholestatic cirrhosis	0.80	1.64	0.63 - 4.33	.313
Acute hepatic failure	0.31	1.357	0.65 - 2.84	.417
Preoperative platelet count (per 10^9/L)	0.001	1.001	0.99 - 1.004	.774
Preoperative serum sodium (mEq/L)	0.02	1.018	0.99 - 1.05	.207
Preoperative serum potassium (mEq/L)	-0.10	.910	0.67 - 1.24	.553
Preoperative serum glucose (mg/dL)	0.001	1.001	0.99 - 1.004	.324
History of esophageal varix ligation	-0.28	0.75	0.48 - 1.20	.232
Portal hypertension	0.13	1.14	0.54 - 2.43	.733
Previous abdominal surgery	0.23	1.26	0.88 - 1.80	.213
Preoperative insulin use	-0.44	0.64	0.26 - 1.61	.345
Preoperative beta-blocker	0.41	1.51	0.68 - 3.35	.307
Preoperative diuretics	0.24	1.27	0.49 - 3.32	.627
Operation time (hour)	0.35	1.42	0.94 - 2.13	.093
Anesthesia time (hour)	-0.36	0.70	0.47 - 1.04	.080
Cold ischemic time (per 30 min)	0.23	1.26	1.01 - 1.58	.045
Warm ischemic time (per 30 min)	0.11	1.12	0.71 - 1.76	.635
GRWR less than 0.8	0.72	2.05	1.02 - 4.15	.045
Crystalloid (per 1 L)	0.04	1.04	0.96 - 1.13	.312
Colloid (per 500 ml)	0.25	1.28	1.08 - 1.52	.004

Intraoperative albumin administration (per 100 ml)	-0.01	0.99	0.91 - 1.01	.895
Red blood cell transfusion (Yes)	0.001	1.001	0.97 - 1.03	.960
Mean central venous pressure (mmHg)	0.01	1.01	0.98 - 1.05	.495
SvO_2 decrease form baseline (per 10%)	0.33	1.39	1.10 - 1.76	.006
Estimated blood loss (per weight)	0.05	1.05	0.89 - 1.23	.576
Intraoperative Epinephrine bolus dose (per 10 mcg)	-0.001	0.69	0.99 – 1.00	.683
Intraoperative dopamine infusion	0.42	1.52	0.98 - 2.36	.060
intraoperative epinephrine infusion	-0.37	0.69	0.23 - 2.09	.510
Intraoperative norepinephrine infusion	0.06	1.06	0.44 - 2.55	.892
Intraoperative continuous renal replacement therapy	-0.30	0.74	0.26 - 2.09	.569
Mean intraoperative femoral arterial pressure (per mmHg)	-0.01	0.99	0.98 - 1.01	.425
Intraoperative mean hemoglobin (g/dL)	0.01	1.01	0.88 - 1.17	.881
Intraoperative mean blood glucose (per 10 mg/dL increase)	0.08	1.08	1.02 - 1.15	.011
Constant	-3.29	0.04		.275

Multivariable logistic regression analysis was performed using all the variables with $p < 0.2$ in univariate logistic analysis.

Nagelkerke R^2 statistic was 0.163. Hosmer and Lemeshow goodness of fit test was not significant at 5% (Chi-square = 10.9, *P*=0.207).

COPD = chronic obstructive pulmonary disease; MELD = model for end-stage liver disease; GRWR=graft-recipient body-weight ratio; SvO_2=mixed venous oxygen saturation.

Supplemental Table S3. Comparison of area under receiver-operating characteristic curve among the different models to predict stage 2 or 3 acute kidney injury.

	Optimal hyperparameter	AUROC (95% CI)	Accuracy	p-value
Logistic regression (LR)		0.72 (0.67-0.76)	0.72	0.005 vs GBM<br>0.021 vs RF<br>0.096 vs DT<br>0.545 vs SVM<br>0.239 vs NB<br>0.600 vs MLP<br>0.137 vs DBN
Gradient boosting machine (GBM)	Maximum depth=4<br>Number of estimators=50,<br>gamma=0.5	0.85 (0.81-0.89)	0.96	0.488 vs RF<br><0.001 vs DT<br>0.003 vs SVM<br>0.002 vs NB<br>0.010 vs MLP<br><0.001 vs DBN
Random forest (RF)	Maximum depth=7<br>Number of estimators=50	0.83 (0.78-0.86)	0.96	<0.001 vs DT<br>0.036 vs SVM<br>0.013 vs NB<br>0.031 vs MLP<br><0.001 vs DBN
Decision tree (DT)	Maximum depth=5<br>Criterion=Gini index	0.59 (0.53-0.64)	0.92	0.329 vs SVM<br>0.520 vs NB<br>0.228 vs MLP<br>0.666 vs DBN
Support vector machine (SVM)	Kernel=linear basis<br>C=0.9	0.68 (0.63-0.73)	0.70	0.673 vs NB<br>0.853 vs MLP<br>0.354 vs DBN
Naive Bayes (NB)	Model=Gaussian	0.64 (0.59-0.69)	0.81	0.511 vs MLP<br>0.808 vs DBN
Multilayer perceptron (MLP)	Number of hidden layers=2<br>Number of nodes in a layer=8	0.69 (0.64-0.74)	0.88	0.098 vs DBN
Deep belief network (DBN)	Number of hidden layers=2<br>Number of nodes in a layer=10	0.63 (0.57-0.67)	0.75	

CI=confidence interval; AUROC=area under receiver operating characteristic curve.
